# Editorial: Experimental & Clinical Epilepsy and Related Comorbidities

**DOI:** 10.3389/fphar.2020.592448

**Published:** 2020-09-11

**Authors:** Mohd. Farooq Shaikh, Ayanabha Chakraborti, Teresa Ravizza, Terence J. O’Brien, Jafri Malin Abdullah

**Affiliations:** ^1^ Neuropharmacology Research Laboratory, Jeffrey Cheah School of Medicine & Health Sciences, Monash University Malaysia, Bandar Sunway, Malaysia; ^2^ Department of Neuroscience, Central Clinical School, Monash University, The Alfred Hospital, Melbourne, VIC, Australia; ^3^ Department of Surgery, School of Medicine, University of Alabama at Birmingham, Birmingham, AL, United States; ^4^ Department of Neuroscience, Istituto di Ricerche Farmacologiche Mario Negri IRCCS, Milan, Italy; ^5^ Brain Behaviour Cluster & Department of Neurosciences, School of Medical Sciences, Hospital Universiti Sains Malaysia, Universiti Sains Malaysia, Kota Bharu, Malaysia

**Keywords:** epilepsy, comorbidities, experimental-animal models, clinical epilepsy, antiepileptics drugs

Epilepsy is a serious neurological condition affecting about 65 million people around the world, and 80% of this population is based in developing countries. Affected individuals have an increased risk for developing various comorbid conditions like cognitive, behavioral, physical, mental, and psychosocial disabilities that can adversely impact the quality of life. Many antiepileptic drugs are found to have a role in aggravating physical and/or psychiatric symptoms. This Research Topic aimed to highlight basic, clinical, and translational research involved in studying epilepsy and associated comorbidities. Translational research is an important aspect of epilepsy research and expected to play a key role in improving the quality of life of the patients. It was also expected to help in understanding the complexity of the condition and its comorbidities. Advances in the discovery of novel potential antiepileptics and their mechanism of action are also being included in this special topic.

This Research Topic has gathered 10 articles, including one brief research report, one mini-review, one hypothesis and theory, two reviews, and five original research contributions from prominent scientists in the field. The collection of papers on this Research Topic provides an up-to-date insight into current knowledge and overview of different approaches in experimental and clinical epilepsy studies as well as related comorbidities. The content of each of these articles is summarized below.

Anti-epileptic drugs (AEDs) are successful in controlling epilepsy but are reported to worsen cognitive status in a significant proportion of patients. The research conducted by Kundap et al. investigated the effectiveness of embelin (EMB), a benzoquinone derived from the plant *Embelia ribes* against pentylenetetrazole (PTZ) induced acute seizures and its associated cognitive dysfunction. This study suggests that EMB suppresses seizure-like behavior through the GABA_A_ receptor pathway and positively influences cognitive functions in zebrafish.

Decreased bone health in epilepsy patients can be commonly attributed to AED intake; therefore, Brady et al. assessed whether bone abnormalities occur in epilepsy in the absence of AEDs by investigating mechanical characteristics and trabecular bone morphology in rats with chronic temporal lobe epilepsy. Their study revealed a lack of overt bone abnormalities in rats with chronic temporal lobe epilepsy in the absence of AED treatment and no differences in mechanical properties of femurs. However, this warrants further studies towards understanding the source of bone abnormalities in epilepsy patients.

Temporal lobe epilepsy is a common and often drug-resistant type of epilepsy in the adult and aging populations. However, kindling studies and kindling-induced behavioral changes in animal models remain scarce. Liu et al. attempt to shed more information in this area using a mouse model of extended hippocampal kindling. The study revealed the mice were impaired in their spatial learning and memory which suggests that the extended hippocampal kindling in middle-aged mice as a model aids in the exploration of epileptogenic mechanisms and comorbidities that could be relevant to temporal lobe epilepsy.

A rare but severe myoclonic epilepsy in infants known as Dravet syndrome (DS) is caused by heterozygous mutations of the *Scn1a* gene. Recently, a glucagon-like peptide-1 (GLP-1) analog, liraglutide has emerged as a potential treatment modality for patients with nervous system diseases. The study by Liu et al. elucidates the neuroprotective role of liraglutide in mouse and cell models of *Scn1a* KO-induced epilepsy. It was found that liraglutide reduces seizure susceptibility and cognitive dysfunction in the DS mouse model with anti-apoptotic and neuroprotective effects in *Scn1a* KO mice and cells.

In a brief research report, Nobili et al. elucidate the outcome of early-chronic carbamazepine (CBZ) administration on both seizure activity and brain damage in methylazoxymethanol-pilocarpine (MP) rat model, whereby occurrence of status epilepticus and subsequent spontaneous seizures induce progressive brain damage. Their data suggest there are no differences between treatment groups, indicating that early-chronic CBZ in food administration to MP rats does not affect convulsive motor seizures.

The identification of an experimental model of absence epilepsy with predictive validity is important for the investigations of its mechanisms and evaluation and justification of experimental treatment alternatives. The review article by van Luijtelaar and van Oijen provides an overview on establishing drug effects through the design of drug evaluation studies, type of rat, traditional and novel electroencephalogram (EEG) variables, monitoring and quantification of rat behavior, limitations in data interpretation, and developments in EEG technology in genetic rat models; WAG/Rij strain and GAERS, for genetic absence epilepsy model.

The review on general early life insults linking to development of epilepsy by Semple et al. describes comprehensive preclinical evidence which demonstrates that early-life immune challenges such as those sustained during early postnatal life, prenatal immune activation, and perinatal injuries influence neuronal hyperexcitability and predispose an individual to epilepsy in later life. The review highlights neuroinflammatory mechanisms, and other indirect variables such as genetics and environment following early-life insults underlie long-term epilepsy risk. Hence, it could provide insight into the development of immunological anti-epileptogenic therapy alternatives.

Autism spectrum disorder (ASD) is a neurodevelopmental disorder with characteristics such as social communication impairments and restricted and repetitive behaviors and interests. Identifying the genetic background could be a vital feature for the diagnosis and treatment of ASD. The research by Lee et al. reports on utilizing next-generation sequencing (NGS) as a tool to analyze multiple genes simultaneously for autism genetics in the laboratory and clinical settings. The research showed VOUS genes are frequently related to ASD or other neurodevelopmental disorders such as epilepsy.

In a mini-review Ogaki et al. discuss the relationships between epilepsy and brain vascular abnormalities such as vascular malformation, blood–brain barrier dysfunction, and excessive angiogenesis. The review highlights the potential role of vascular endothelial growth factor (VEGF) and VEGF signaling as a therapeutic strategy by modulating the structure and function of the neurovascular unit in the epileptic brain.

The gut–brain-axis influence poses a hypothesis that links both epilepsy and associated comorbidities. The review by Shaikh et al. serves to highlight the possible influence of the gut–brain-axis in the manifestation of depressive symptoms in epilepsy. There is indirect evidence that revealed some specific bacterial strains that might cause depression in epilepsy which suggests a correction of this dysbiosis through probiotics supplementation might be beneficial in treating both epilepsy and related depression.

The diversity of these papers has contributed greatly to the library of basic research and clinical studies in the field of clinical epilepsy and associated comorbidities. We are grateful and hopeful that these papers would benefit and inspire other researchers to work and advance this exciting research field. Further studies that contribute towards a better understanding of the pathogenesis of such conditions can potentially lead to novel development of effective and safer treatment options for clinical epilepsy and associated comorbidities.

## Author Contributions

MS took the initiative for the editorial write-up. AC, TR, TO’B, and JA also contributed to revising and proofreading. All authors contributed to the article and approved the submitted version.

## Conflict of Interest

The authors declare that the research was conducted in the absence of any commercial or financial relationships that could be construed as a potential conflict of interest.

